# A multiplexed RT-PCR assay for nanopore whole genome sequencing of Tilapia lake virus (TiLV)

**DOI:** 10.1038/s41598-023-47425-w

**Published:** 2023-11-20

**Authors:** Jérôme Delamare-Deboutteville, Watcharachai Meemetta, Khaettareeya Pimsannil, Pattiya Sangpo, Han Ming Gan, Chadag Vishnumurthy Mohan, Ha Thanh Dong, Saengchan Senapin

**Affiliations:** 1 WorldFish, Bayan Lepas, Penang Malaysia; 2 Fish Health Platform, Center of Excellence for Shrimp Molecular Biology and Biotechnology (Centex Shrimp), Faculty of Science, Mahidol University, Rama VI Rd., Bangkok, 10400 Thailand; 3 Patriot Biotech Sdn Bhd, Bandar Sunway, 47500 Subang Jaya, Selangor Malaysia; 4 School of Environment, Resources and Development, Asian Institute of Technology, Pathum Thani, 12120 Thailand; 5 National Center for Genetic Engineering and Biotechnology (BIOTEC), National Science and Technology Development Agency (NSTDA), Pathum Thani, 12120 Thailand

**Keywords:** Viral infection, Genome assembly algorithms, Data processing, Phylogeny, DNA sequencing, Next-generation sequencing, Bioinformatics

## Abstract

Tilapia lake virus (TiLV) is a highly contagious viral pathogen that affects tilapia, a globally significant and affordable source of fish protein. To prevent the introduction and spread of TiLV and its impact, there is an urgent need for increased surveillance, improved biosecurity measures, and continuous development of effective diagnostic and rapid sequencing methods. In this study, we have developed a multiplexed RT-PCR assay that can amplify all ten complete genomic segments of TiLV from various sources of isolation. The amplicons generated using this approach were immediately subjected to real-time sequencing on the Nanopore system. By using this approach, we have recovered and assembled 10 TiLV genomes from total RNA extracted from naturally TiLV-infected tilapia fish, concentrated tilapia rearing water, and cell culture. Our phylogenetic analysis, consisting of more than 36 TiLV genomes from both newly sequenced and publicly available TiLV genomes, provides new insights into the high genetic diversity of TiLV. This work is an essential steppingstone towards integrating rapid and real-time Nanopore-based amplicon sequencing into routine genomic surveillance of TiLV, as well as future vaccine development.

## Introduction

Tilapia lake virus disease (TiLVD) is a highly contagious viral disease that affects tilapia, an important and affordable fish protein source produced in aquaculture globally. TiLV has rapidly spread since, now reported in over 18 countries^1–4^, posing a significant threat to tilapia production and the livelihoods of farmers who rely on tilapia farming for income and food security^5^. To mitigate the introduction and spread of TiLV and its impacts, it is imperative to implement surveillance, improved biosecurity measures, farming practices and continuous development of effective diagnostic and rapid sequencing methods.

The TiLV genome consists of 10 segments that complicate its genome sequencing process thus precluding high mass-scale genome sequencing efforts to be undertaken. The first TiLV genome was sequenced using a shotgun transcriptome approach on an Illumina sequencing platform^6^. The genomes of TiLV were also sequenced using the Sanger sequencing technique^7,8^. Recently, a similar approach using shotgun metagenomics was used to generate the near complete genome of a TiLV isolate causing mass-mortality in tilapia farmed in Bangladesh^9^. Shotgun metagenomics involved the random sequencing of all RNA fragments, i.e., TiLV-positive tilapia liver samples without any enrichment of mRNA; followed by bioinformatics analysis to identify and assemble the 10-segments of TiLV genome present in the sample. This approach is not scalable as the RNA library preparation cost is higher and most of the sequencing data will belong to the host, requiring high sequencing depth to successfully assemble the TiLV-derived contigs. To address these challenges and improve sequencing effectiveness, some approaches have been explored. One such approach involves the propagation of viruses in cell culture prior to sequencing^10^. Additionally, enrichment through single RT-PCR amplification of the TiLV 10 segments before subjecting them to Illumina sequencing has also been employed^3^. Nevertheless, the use of Illumina technology necessitates a significant investment in infrastructure, which hinders rapid on-site deployment and real-time sequencing.

In recent years, Oxford Nanopore Technologies (ONT) have become more commonly used to sequence part(s) or whole genomes of pathogens affecting aquatic animals^11^. Nanopore sequencing offers several advantages, including high throughput, portability, cost-effectiveness, and real-time sequencing, which can greatly facilitate the detection and sequencing of viral genomes in remote locations^12^. Rapid amplicon-based reverse transcription polymerase chain reaction (RT-PCR) assays coupled with Nanopore technologies can provide a sensitive and specific means of detecting and genotyping TiLV based on segment 1 in field samples. This approach allows for fine epidemiological surveillance and timely management and control of outbreaks^13^. While Nanopore has been used to sequence the genomes of non-segmented fish viruses such as infectious spleen and kidney necrosis virus (ISKNV)^12^, salmonid alphavirus (SAV) and segment 5 and 6 of infectious salmon anaemia virus (ISAV)^11^, it has not yet been applied to the TiLV genome.

In this study, for the first time, we designed a new method for TiLV whole genome sequencing using singleplex and multiplex amplicon-based RT-PCR protocols coupled with Minion Nanopore sequencing. These novel tools enable real-time diagnosis and characterization of TiLV genomes, thereby facilitating improved surveillance and effective control measures in tilapia aquaculture.

## Methods

### Ethics declarations

No animal ethical approval was required for this study as it involved the utilization of archived preserved fish tissue samples. These tissues were obtained from experimentally infected fish and constituted our archived samples from a previous study conducted by our research group. All methods were performed in accordance with the relevant guidelines and regulations. The animal ethics committee of the Faculty of Science at Mahidol University had approved all the experimental protocols of that previous study (ethics approval #MUSC62-017-481). For further details, please refer to the corresponding publication available at Dong et al. 2020^14^. Additionally, the tissues obtained from naturally infected fish used in this study were previously provided to our laboratory, as described in our publication by Taengphu et al. 2022^15^.

### Primer design

Primer sequences targeting all 10 complete genome segments of TiLV were manually designed with reference to the TiLV genome of the Israel strain Til-4-2011 (GenBank accession no. KU751814 to KU751823)^6^ (Table 1). The primer sequences were carefully selected at the outermost regions of the 5′ and 3′ terminal ends, enabling the amplification of full-length genomic segments, thereby ensuring maximal preservation of genetic information. In addition to our primary objective of achieving full-length amplification, we followed general criteria for PCR primer design as outlined by ThermoFisher Scientific (2019)^16^. These criteria encompassed a primer length of 22–25 oligonucleotides in this study, ensuring that each primer pair had similar melting temperature (Tm) and %GC content.

**Table 1:** TiLV primers designed in this study were based on the TiLV genome isolate of the Israeli strain Til-4-2011 (GenBank accession no. KU751814 to KU751823).

Target gene	Sequence (5′ → 3′)	Ta (°C) used in sPCR	Ta (°C) used in mPCR	Size (bp)
Segment 1	CS1F; GCAAATATTTCTCTCATTCGCCTAT CS1R; CCAAACGTTATCTCTTAATTACGCA	55	52	1641
Segment 2	CS2F; GCAAATCTTTCTCATTATTACCATA CS2R; CCAAATTTTACTCTCTATTACCAAA	55	52	1471
Segment 3	CS3F; GCAAATTTTTCCCATAATCCTCTAT CS3R; CCAAATATTACCCCTTAATCCTTAA	55	52	1371
Segment 4	CS4F; GCAAATCTTTCTCCAATTACCGTCT CS4R; CCAAAGTTTACTCCTATTACCCAGA	50	52	1250
Segment 5	CS5F; GCAAATTTTACTCTTTTTCTCAGTT CS5R; CCAAATGTTTCTCTTATCTCAGACT	58	52	1099
Segment 6	CS6F; GCAAATATTTCTCTCAATCAAGCAC CS6R; CCAAATTTTACCTCTCGCATGCATT	60	60	1044
Segment 7	CS7F; GCAAATCTTTCTCTCATGCTACCAT CS7R; CCAAATTTTACTCTCTTTGCATTGC	60	60	777
Segment 8	CS8F; GCAAATTTTTCTCATCATTACACAA CS8R; CCAAATATTACCTCATCTACACTAA	58	52	657
Segment 9	CS9F; GCAAATCTTTCTCACGTCCTTAAAG CS9R; CCAAATTTTACTCACAAGTCCGATT	60	60	548
Segment 10	CS10F; GCAAATCTTTCCCTCTGACACC CS10R; CCAAATTTTAACCCTACTAACACCA	60	60	465

### Samples, total RNA extraction, and TiLV quantification

RNA templates (N = 10) for the amplification and analysis of the TiLV genome sequence were prepared from various sources, including tissues of TiLV-infected Nile tilapia (*Oreochromis niloticus*) and red tilapia (*Oreochromis* spp.), TiLV isolates propagated in E-11 cell culture, and a concentrated water sample from a tilapia rearing river (Table 2). RNA from fish tissues (liver, kidney, spleen, and/or brain) was extracted using Trizol reagent (Invitrogen), while RNA from TiLV-infected E-11 cell culture was isolated using MagTec™ ViroNA Nano-magnetic beads for DNA/RNA virus isolation (Bioentist), following the manufacturer’s instructions. The virus in the river water sample was concentrated using the iron flocculation method and then filtered through a 0.4-μm pore size filter using a vacuum pump^15^. The filters that trapped the flocculate were subsequently used for nucleic acid extraction with the Patho Gene-spin DNA/RNA extraction kit (iNtRON Biotechnology). The obtained nucleic acids were quantified using spectrophotometry, measuring absorbance at OD_260_ nm and OD_280_ nm. TiLV quantification by probe-based qPCR assays of the 10 samples were performed based on segment 9^15^ and segment 1 (this study) (Supplemental Table 1).

**Table 2:** Background information of TiLV strains reported in study as well as strains with publicly available genomes.

#	Sequence name (strain)	Country of origin	Collection date	Fish host	Tissues	Accession number	Reference	Sequencing Technology
1	B1-1_m	TH	2021	Red tilapia	L, K, S	SRX20019084	This study	Nanopore
2	B1-2_m	TH	2021	Red tilapia	L, K, S	SRX20019085	This study	Nanopore
3	FM2_m	TH	2019	Nile tilapia	L, K, S	SRX20019089	This study	Nanopore
4	Nk_m	TH	2019	Nile tilapia	L, B	SRX20019090	This study	Nanopore
5	A1-2_m	TH	2021	Red tilapia	L, K, S	SRX20019091	This study	Nanopore
6	A1-3_s	TH	2021	Red tilapia	L, K, S	SRX20019096	This study	Nanopore
7	A1-3_m	TH	2021	Red tilapia	L, K, S	SRX20019092	This study	Nanopore
8	D1-2_s	TH	2021	Red tilapia	L, K, S	SRX20019086	This study	Nanopore
9	D1-2_m	TH	2021	Red tilapia	L, K, S	SRX20019093	This study	Nanopore
10	Ri_m	TH	2020	Nile tilapia	L	SRX20019094	This study	Nanopore
11	Cell_line_s	TH	2019	Red tilapia	E-11 cells	SRX20019088	This study	Nanopore
12	Cell_line_m	TH	2019	Red tilapia	E-11 cells	SRX20019095	This study	Nanopore
13	RiverWater_s	TH	2021	Red tilapia (water)	na	SRX20019087	This study	Nanopore
14	IL-2011 (Til-4–2011)	IL	1 May 2011	Hybrid tilapia	na	KU751814–823	^27^	Illumina
15	TH-2013	TH	Jan 2013	Nile tilapia	Fe	MN687685-695	^28^	Sanger
16	TH-2014	TH	Aug 2014	Nile tilapia	Fr	MN687695-704	^8^	Sanger
17	TH-2015	TH	Aug 2015	Nile tilapia	Fi	MN687705-714	^8^	Sanger
18	TH-2016-CU	TH	Dec 2016	Nile tilapia	Ju	MN687715-724	^8^	Sanger
19	TH-2016-CN	TH	Dec 2016	Red tilapia hybrid	Fi	MN687725-734	^8^	Sanger
20	TH-2016 (TV1)	TH	2016	Red tilapia	na	KX631921–930	^7^	Sanger
21	TH-2017	TH	2017	Nile tilapia	na	MN687735-744	^8^	Sanger
22	TH-2018-N	TH	Jul 2018	Red tilapia	Fi	MN687745-754	^8^	Sanger
23	TH-2018-K	TH	Aug 2018	Nile tilapia	Ju	MN687755-764	^8^	Sanger
24	TH-2018 (WVL18053-01A)	USA	Apr 2018	Nile tilapia		MH319378–387	^10^	Illumina
25	TH-2019	TH	Feb 2019	Nile tilapia	Fi	MN687765-774	^8^	Sanger
26	EC-2012 (EC-2012)	EC	2012	Nile tilapia	na	MK392372–381	^29^	Illumina
27	PE-2018 (F3-4)	PE	2018	Nile tilapia	na	MK425010–019	^30^	Sanger
28	US-2019 (WVL19031-01A)	US	2018	Nile tilapia	na	MN193513–522	Unpublished*	Illumina
29	US-2019 (WVL19054)	US	2019	Nile tilapia	na	MN193523–532	Unpublished*	Illumina
30	BD-2017	BD	2017	Nile tilapia	na	MN939372–381	^9^	Illumina
31	BD-2019-E1	BD	2019	Nile tilapia	na	MT466447-456	^31^	Sanger
32	BD-2019-E3	BD	2019	Nile tilapia	na	MT466457-466	^31^	Sanger
33	BD-2017–181	BD	2017	Nile tilapia	na	MT466437-446	^31^	Sanger
34	IND-2018	IND	2017	Nile tilapia	na	MZ297923-932	Unpublished*	Illumina
35	VN (HB196-VN-2020)	VN	2020	Nile tilapia	na	ON376572-581	^3^	Illumina; Sanger
36	VN (RIA2-VN-2019)	VN	2019	Nile tilapia	na	ON376582-591	^3^	Illumina; Sanger

Country of origin: TH, Thailand; IL, Israel; EC, Ecuador; PE, Peru; US, USA; BD, Bangladesh; IND, India; VN, Vietnam.

Fish host: Red tilapia, *Oreochromis* sp.; Nile tilapia, *Oreochromis niloticus*; Hybrid tilapia *Oreochromis niloticus* × *Oreochromis aureus;* L, Liver; K, Kidney; S, Spleen; B, Brain; Fe, fertilized eggs; Fr, fry; Fi, fingerlings; Ju, juveniles; na, info not available; *, direct submission. Sequence name for samples #1–13: _s (singleplex PCR), _m (multiplex PCR) with their corresponding SRA accession numbers. The links to the BioSamples can be found in the data availability section.

### Development of singleplex one-step RT-PCR for the enrichment of TiLV genome

The efficiency of the designed TiLV primers and their optimal annealing temperatures (Ta) were investigated by one-step gradient RT-PCR assays with the range of Ta from 50 to 60 °C. Singleplex RT-PCR (sPCR) reaction mixture of 25 µL were prepared and subjected to amplifications as outlined in Supplemental Table 1 Amplified products of the singleplex RT-PCR (sPCR) were analyzed by agarose gel electrophoresis. Four RNA templates were used in this assay (Table 2).

### Development of multiplex RT-PCR to streamline the PCR enrichment of TiLV genome

Two multiplex PCR (mPCR) reactions were developed to reduce the number of PCR reactions from 10 to only two reactions per sample. The primers were divided into two sets based on their annealing temperatures similarity, and we also considered the separability of product sizes on agarose gel electrophoresis. Initial trials with different primer mixes and amplification conditions were conducted (data not shown). Based on the results, reaction 1 employs primers for segment 1, 2, 3, 4, 5 and 8 with Ta at 52 °C while reaction 2 uses primers for segment 6, 7, 9 and 10 with Ta at 60 °C. Detailed reaction mixtures and amplification conditions are shown in Supplemental Table 1. Then, various PCR conditions were further tested by varying the dNTPs (200–500 nM), MgSO_4_ (1.6–1.8 mM), enzyme (1–2.5 μl) and primer concentrations (100–300 nM) to obtain optimal PCR outcomes. Nine RNA templates were used in this assay (Table 2).

### Nanopore sequencing

PCR products from the singleplex (15 µl of each 10 PCR reactions) and the multiplex (40 µl of each 2 PCR reactions) were pooled followed by PCR clean up using NucleoSpin Gel and PCR Clean-up column (Macherey–Nagel) and quantification with Qubit dsDNA Broad Range kit (Invitrogen). Approximately 250 ng of the purified and pooled amplicons was used as the template for library preparation using the native barcoding expansion 1–12 kit (EXP-NBD104) according to the manufacturer’s instructions. The prepared library was loaded onto a R9.4.1 Flongle and sequenced for 24 h. Basecalling of the fast5 raw signals used Guppy v4.4.1 in super accuracy mode to generate the fastq sequences for subsequent bioinformatics analysis.

### Reference-based genome assembly of TiLV samples

Raw reads were quality- and length-filtered using NanoFilt (qscore > 9 and length > 250 bp). The raw and filtered read statistics were generated using seqkit v.2.1.0. Reference-based genome assembly of the TiLV was performed according to the ARTIC pipeline (https://github.com/artic-network/fieldbioinformatics)^17^. This pipeline is an open-source software that integrates a series of tools for base-calling, quality control, read trimming, reference-based mapping, variant calling, consensus sequence generation, and annotation. Briefly, the filtered reads were aligned to the reference TilV genome using Minimap2 v2.17^18^ followed by variant calling using Medaka (r941_min_sup_g507) (https://github.com/nanoporetech/medaka). The variants identified were subsequently filtered based on several criteria, including the quality score, depth of coverage, strand bias, and frequency of occurrence. In addition, genomic regions with read depth of lower than 20× were masked prior to generating the final consensus sequence for each sample. Each assembled viral segment from each sample was analyzed with QUAST v5^19^ to calculate the percentage of the assembled viral genome that is represented by gaps (Ns), providing insights into the PCR and pooling efficiency.

### Phylogenetic analysis

The assembled viral segments with less than 20% gap were selected and combined with publicly available TiLV genomes for phylogenetic analysis (Table 2). The DNA sequences of the viral genome segments from each sample were extracted and grouped based on their segment number followed by alignment with MAFFT v8 (-adjustdirection -maxiterate 1000 -localpair)^20^. All 10 individual alignments were subsequently concatenated and used to reconstruct a maximum likelihood tree using FastTree 2^21^. The resulting tree was visualized and annotated using FigTree v1.4.4 (http://tree.bio.ed.ac.uk/software/figtree/).

## Results

### Ten primer pairs for the recovery of complete TiLV genome from various isolation sources

**Figure 1:**
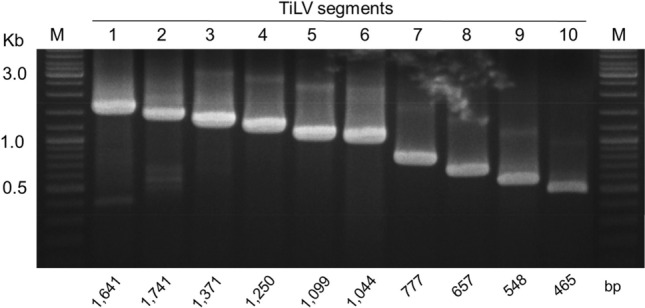
Gel electrophoresis (original photo) results of one-step RT-PCR amplification of 10 genomic segments of TiLV. Representative results from sample D1-2 are shown. A 1% agarose gel was used to visualize the PCR products, with expected band sizes indicated at the bottom of the gel. M, DNA marker (New England Biolabs). The original unlabeled image can be found in the supplemental materials.

A total of 10 primer pairs were designed with their PCR condition optimized (Table 1 and Supplemental Table 1) to amplify the complete segment of one of the ten TiLV genomic segments. Intact and specific band corresponding to the respective size of the TiLV genomic segments were successfully obtained when the total RNA extracted from TiLV-infected tilapia, TiLV-infected E-11 cell line, and concentrated river water sample were used as the template for RT-PCR (Fig. 1). However, the PCR band intensity for segment 4 (1250 bp) of the water samples is substantially lower compared to the other segments, requiring another round of PCR (Table 3, Supplemental Table 1).

**Table 3:**
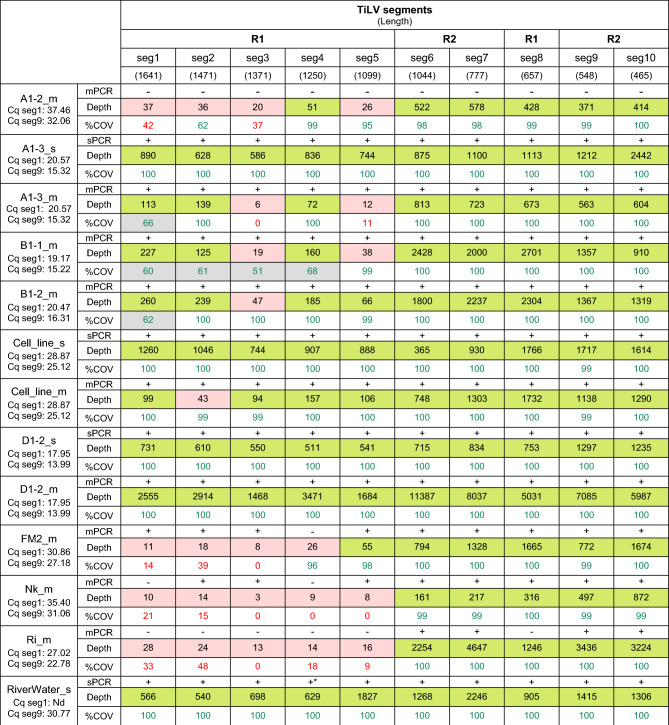
PCR outcome, sequencing, and alignment statistics of 10 individual TiLV segments for each sample used in this study.

Viral segments (seg1-5 and seg8) and (seg6, 7, 9, 10) were amplified in two multiplex reactions, reaction 1 (R1) and reaction 2 (R2), respectively. The Cq values from the qPCR detection of segment 1 (Cq seg1) and segment 9 (Cq seg9) were shown below each sample. +/− in the sPCR (singleplex PCR) and mPCR (multiplex PCR) row indicates presence (+) or absence (−) of visible PCR band representing the respective viral segment. %COV (% coverage) indicates the percentage of bases in the assembled contig that consists of a non-ambiguous base; %COV < 50 in red and > 50 in green; Depth indicates sequencing depth (or read depth); Depth < 50 are colored in orange and > 50 in green.

+ , detectable; − , undetectable; + *, detectable from re-amplification; Nd, Not done.

### A streamlined two-tube multiplex RT-PCR for TiLV genome amplification

**Figure 2:**
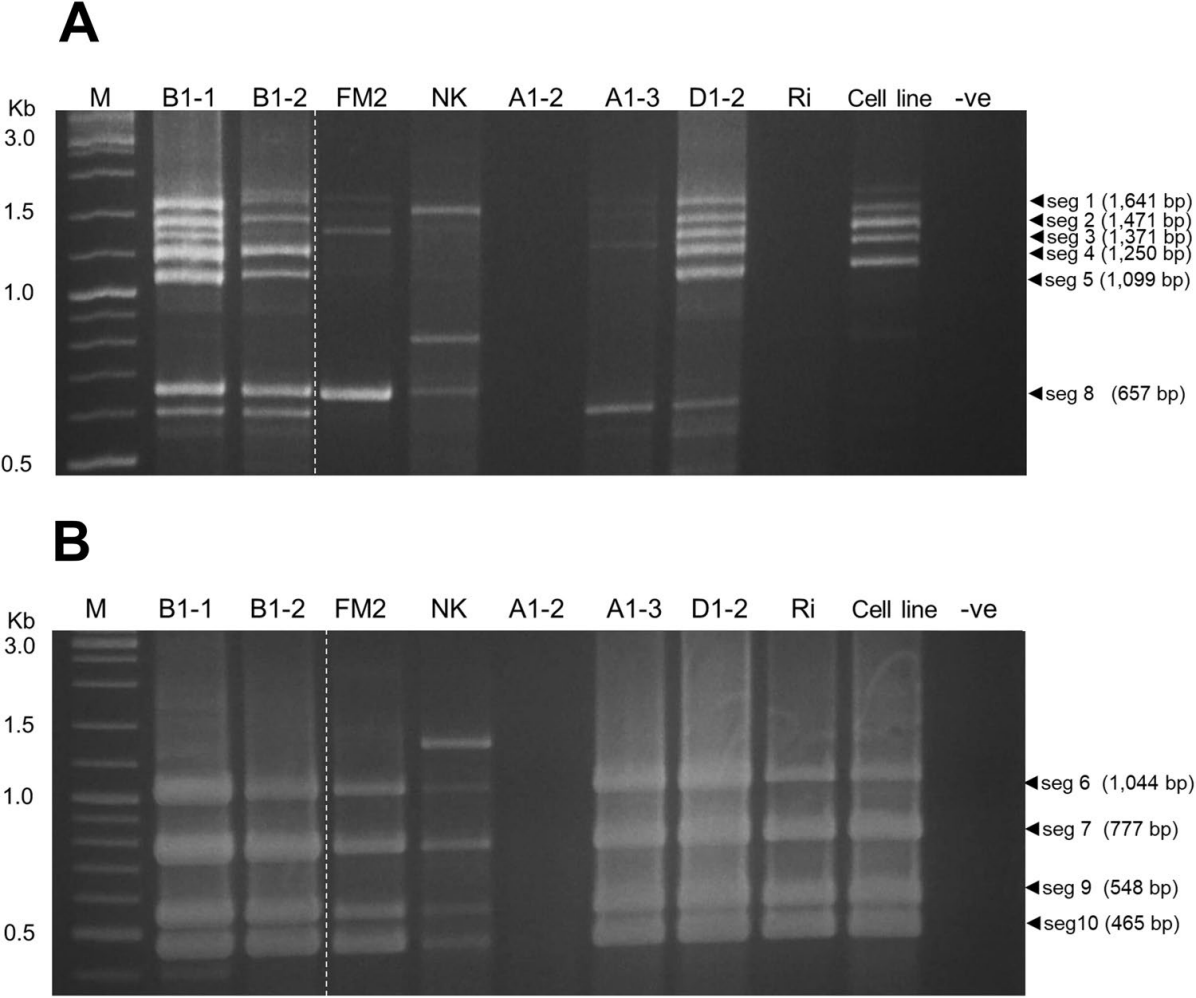
Amplification results of multiplex PCR (mPCR) for TiLV segments. Two separate reactions (Reaction#1 and Reaction#2) were used to amplify 10 TiLV segments. Reaction#1 amplified segments 1, 2, 3, 4, 5, and 8 (**A**), while Reaction#2 amplified segments 6, 7, 9, and 10 (**B**). A 2-log DNA marker (New England Biolabs) was used to visualize the PCR products. −ve, no template control. Codes of samples are listed in Table 2. The white dash lines indicate where two images were connected since one lane (Lane X) was excluded from the original gel photo (Supplemental Fig. 3). The original unlabeled image can be found in the supplemental materials.

To minimize the risk of human error associated with handling multiple singleplex PCR reactions (10 per template), and to reduce chemical costs, a two-tube multiplex RT-PCR was designed (Supplemental Table 1). The addition of MgSO_4_ (increased magnesium ion concentration) was crucial for improved sensitivity (stronger band intensity) while an increase in dNTP concentration does not improve PCR efficiency (Supplemental Fig. 1). In addition, increasing the amount of RT/Taq enzyme mix was also shown to slightly improve over band intensity (Supplemental Fig. 1). As a result, the concentration of MgSO_4_ and RT/Taq enzyme mix was increased in further multiplex RT-PCR assays (Supplemental Fig. 2). After applying the final mPCR conditions to the 10 RNA templates, it was not surprising to observe that samples with high TiLV loads (as determined by qPCR assays) (Table 2) produced the expected six bands and four bands in mPCR reaction 1 and 2, respectively (Fig. 2, Table 3). These samples included A1-3, B1-1, B1-2, D1-2, FM2, and the cell line. In contrast, samples NK and Ri, which had lower TiLV loads, showed some missing amplicons, while sample A1-2 exhibited no observable bands in either multiplex RT-PCR reactions (Fig. 2, Table 3). It is important to note that mPCR reaction 1 is less sensitive than mPCR reaction 2 (Fig. 2) and inconsistently produces observable bands when the Cq value of the tested sample exceeds 19.

### Rapid and on-site sequencing of the TiLV genome using oxford nanopore

A total of 413,379 demultiplex raw reads with an accumulative length of 238,983,973 bp were generated from the Flongle sequencing runs (Supplemental Table 2). After filtering (qscore > 9, length > 250 bp), only 194,564 reads (147,415,018 bp) remain. On average more than 35% data reduction was observed across the samples with sample A1-2 showing the largest reduction (67.7%, from 28 to 9.2 Mb) in the amount of usable data. Overall, the Arctic-based reference genome assembly could successfully assemble the viral segments 6,7,8,9,10 for all samples with more than 99–100% completeness except for samples A1-2_m and Nk_m that showed only a slightly lower completeness of 98% for segments 6 and 7 (Table 3). On the contrary, several segments from the first set of multiplex RT-PCR showed reduced completeness (high % of gaps in sequence) particularly for samples with high Cq. The reduced completeness is a direct result of the low read depth (< 20) observed for the viral segments in the respective samples. Generally, any viral segment with a read depth of more than 50× will produce a highly complete assembly that can be used in subsequent analysis (Table 3).

### High phylogenetic diversity among Thai TiLV strains

**Figure 3:**
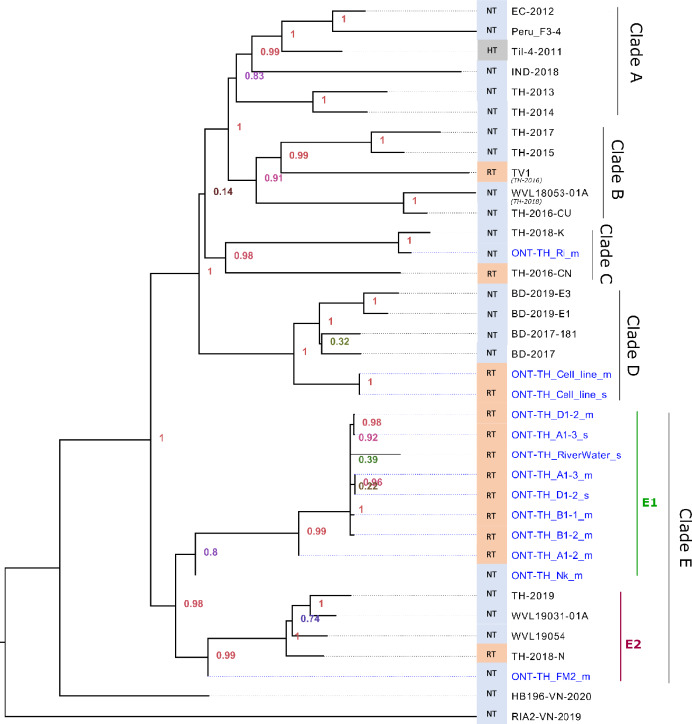
Maximum likelihood tree showing the evolutionary relationships of TiLV strains analyzed in this study. Thirteen samples (10 unique strains) with ONT-TH prefix and publicly available genomes were used. The blue colored tip labels indicate the TiLV strains reported in this study. SH-like local support values and branch length indicate the number of substitutions per site. NT: Nile tilapia; RT: Red tilapia; HT: Hybrid tilapia.

The total alignment length after the concatenation of 10 individually aligned TiLV viral segments is 10,396 bp. Using a midpoint rooting approach, multiple clades with high SH-like support values were observed in the maximum likelihood tree (Fig. 3). Nanopore-sequenced samples from either the pooled singleplex (N = 4 templates) or multiplex amplicons (N = 9 templates) were always placed in the same cluster, consistent with their identical sample origin (Table 2). This observation suggests that accurate genome sequences can be obtained using either singleplex or multiplex amplicon enrichment methods. TiLV strains from Peru, Ecuador, Israel, and India were clustered together and this subclade subsequently formed a sister group with slightly lower support with two earlier TiLV strains from Thailand isolated in 2013 and 2014 to form Clade A (Fig. 3). Clade B consisting entirely of Thai TiLV strains from 2015 to 2016 formed a sister group with Clades A. However, a majority of Thai TiLV strains that were reported in 2018 onwards showed yet another distinct clustering as indicated by their phylogenetic placement in Clades C and E with most of the sequences reported in this study belonging to subclade E1. On the other hand, subclade E2 consists of a mixture of Thai and USA TiLV strains. The currently sampled Bangladeshi TiLV strains consist of only single clade despite being isolated 2 years apart (2017 and 2019) while the Vietnamese strains are highly divergent even between themselves (Pairwise nucleotide similarity of only 92%), possibly representing novel strains of TiLV.

## Discussion

Nanopore sequencing technology is known for its capability to sequence a broad range of DNA inputs including non-target bands. To ensure the specificity of our study, we employed an amplicon-based approach with specific primers. However, it's important to note that this approach does not eliminate completely the potential for sequencing non-target DNA bands. To address this issue, we implemented an *in-silico* alignment of the generated reads to the TiLV virus reference. This strategic step allowed us to effectively filter out non-target reads and data, thereby enhancing the accuracy and specificity of our sequencing results.

In this study, we report the successful recovery of the complete TiLV genome using a novel approach that combines singleplex PCR and multiplex PCR and Nanopore amplicon sequencing. The approach accommodates a range of biological resources, including fish tissues, water samples, and cell cultures, with appropriate sample preparation steps tailored to the nature of each sample type. Our findings indicate that mPCR is particularly effective for samples with high TiLV loads. Therefore, we recommend utilizing mPCR for heavily infected TiLV samples, while sPCR can be employed for lightly infected samples. Notably, since the maximum length of the viral segment is 1641 bp, our approach obviates the need for a PCR-tiling strategy typically used for recovering large non-segmented viruses such as ISKNV and SAR-CoV-2^12,22^. Moreover, our method offers the added advantage of visualizing PCR efficiency and specificity for each viral segment on a gel, as each fragment has a different size.

Our current multiplex PCR appears to show lower efficiency for Multiplex reaction 1, particularly in samples with high Cq values. The variation in sequencing depth among different segments may be attributed to amplicon input and amplicon size and can result in reduced completeness and gaps in the consensus. It is evident that Multiplex reaction 1 predominantly amplifies larger segments of the TiLV genome, specifically segment 1–5. Larger amplicon sizes can pose challenges in terms of PCR sensitivity, particularly when dealing with samples featuring lower viral loads. To improve the multiplex RT-PCR amplification uniformity and efficiency, the performance of poorly performing primers can be enhanced by optimizing their concentrations or adjusting their annealing temperature by altering their sequence length.

Nevertheless, it remains unknown whether individual genomic segments of TiLV exhibit variations in expression levels and timing, akin to the patterns observed in other viruses^23,24^. Additionally, the Multiplex set 1 reaction, which amplifies TiLV genomic segments 1–5 and 8, can be further split into two pools (e.g., 1A and 1B) that will amplify an average of three viral segments each. In addition, the use of a more processive High-Fidelity Taq polymerase such as Q5 from New England Biolabs that was currently used for high-degree multiplex tiling PCR of the SAR-CoV19 and ISKNV viral genomes is also worth exploring^12,17^. It is also worth noting that despite the absence of visible bands for some of the samples, partial or even near-complete genome assembly was still attainable using our sequencing pipeline. It is possible that the amount of PCR product is below the detection limit of gel-staining dye at its loading concentration although it is in fact present in the samples as revealed from sequences information. To streamline future work in high throughput sequencing of TiLV using this approach, gel visualization may be skipped once a lab can consistently reproduce the PCR outcome with evidence from sequencing data.

Nanopore sequencing is an attractive approach for viral amplicon sequencing due to its portability, convenience, and speed^13^. Our method, which utilizes Nanopore sequencing, eliminates the need for additional fragmentation steps, allowing motor proteins to be directly ligated to amplicons for native sequencing. On the same day, tens of samples can be prepared and sequenced, and the low computing requirements of the ARTIC protocol enable swift genome assembly on a laptop computer, without requiring access to a dedicated server. To further streamline TiLV genome sequencing on the Nanopore platform, we suggest designing multiplex primers that incorporate a partial adapter suitable for Nanopore sequencing^25^. This enables cost-effective PCR-based barcoding that is both efficient and scalable. In cases of low data output, samples can be re-pooled and sequenced on a separate flow cell to achieve the necessary sequencing depth for genome assembly.

By utilizing R9.4.1 sequencing chemistry with super accuracy mode and implementing the ARTIC pipeline, we successfully recovered TiLV genomes that are highly suitable for phylogenetic inference. Our study revealed the presence of TiLV in both fish and environmental water samples from the same farm, which clustered together in Clade E1. Our approach, combining the previously reported water sample TiLV concentration method^15^ with a singleplex RT-PCR amplicon-based Nanopore sequencing strategy, allowed for direct recovery of TiLV genomes from water samples. This innovative method has significant implications for non-lethal, environmental DNA/RNA monitoring, as it eliminates the need for sacrificing fish for genomic analysis. The potential applications of our innovative approach extend to TiLV detection and genome sequencing in fish samples across wider geographical areas. Additionally, for cell culture samples, our approach can serve both as a confirmatory diagnosis tool and allow us to investigate genetic stability of TiLV in relation to potential downstream events, such as viral virulence and genetic changes over time. To gain a more comprehensive understanding of TiLV genetic variations and dynamics, further research is essential, involving the collection of additional samples and more extensive comparative studies.

Our analysis suggests that, in addition to country of origin, the genetic background of the hosts may also contribute to the clustering patterns observed within Clade E. With few exceptions, our results indicate phylogenetic grouping of Thai TiLV strains (E1: red tilapia, E2: Nile tilapia), suggestive the likelihood of multiple introductions into the country or rapid viral evolution. The presence of the Thai isolates in multiple clusters indicates a significant genetic diversity within the virus. RNA viruses are known for their high mutation rate attributed to the absence of proofreading ability in RNA polymerases^26^, allowing them to undergo rapid evolutionary changes.

Furthermore, our findings based on the current genomic sampling contradict the initial hypothesis previously put forth on Tilapia trade movement, which was based on a small genome-based phylogenetic tree with limited supported clustering of Bangladeshi and Thai TiLV strains^9^. Specifically, we found no grouping of Thai strains within the Bangladesh clade (Fig. 3, Clade D), thereby reducing support for the previously proposed hypothesis.

Although viral whole genome sequencing of TiLV is now technically feasible, the current representation of its genome in public databases is limited, making it difficult to infer its evolutionary relationships. Given the significant impact of TiLV on the tilapia aquaculture industry, there is a critical need for more robust genomic surveillance to facilitate better management and tracking in relevant regions. Our method can be used in future studies to generate more representative genomes from Vietnam. Our proposed multiplex PCR Nanopore-based amplicon sequencing approach offers a promising solution, as it enables cost-effective and high-throughput sequencing of TiLV virus genomes. This strategy is poised to revolutionize the field of advanced diagnostics and surveillance of multiple pathogens concurrently from biological samples of animals as well as environmental DNA/RNA of pathogens in water, within a single assay. This strategy eliminates the need for separate reactions and reduces the overall cost and time required for sequencing multiple samples. We anticipate that our approach will provide a valuable resource for ongoing efforts to understand the molecular epidemiology and evolution of TiLV, with important implications for disease control and prevention (e.g., vaccination). The implementation of our method in resource-limited regions is expected to face several challenges, e.g., limited access to finance and skilled labor, procurement, and logistical difficulties. As a result, the primary focus should be on enhancing the capacity of local operators by fostering collaboration with local institutions. This collaborative effort will leverage both existing and new resources to kickstart pilot programs aimed at refining the implementation strategy within real-world settings.

### Supplementary Information

Supplementary Information 1.

Supplementary Information 2.

Supplementary Information 3.

Supplementary Information 4.

Supplementary Information 5.

Supplementary Figure 1.

Supplementary Figure 2.

Supplementary Figure 3.

Supplementary Tables.

## Data Availability

The datasets generated during and/or analyzed during the current study that support our findings are available in the National Center for Biotechnology Information (NCBI) repository at the following persistent web links: demultiplexed FastQ files for all ten samples can be found under BioProject PRJNA957495 with the corresponding BioSample accession SAMN34257318 (B1-1), SAMN34257319 (B1-2), SAMN34257320 (FM2), SAMN34257321 (Nk), SAMN34257322 (A1-2), SAMN34257323 (A1-3), SAMN34257324 (D1-2), SAMN34257325 (Ri), SAMN34257326 (Cell_line), SAMN34257327 (RiverWater). The Sequence Read Archive (SRA) accession numbers for all samples can be found in Table 2. The intermediary files generated during the bioinformatic analyses are publicly available in the Zenodo.org dataset (https://zenodo.org/record/7851622).

## Code availability

The Linux scripts used to generate raw fastq files, assembled genomes and phylogenetics tree are publicly available in the Zenodo.org dataset (https://zenodo.org/record/7851622).
