# Supplementary figures and images for: A multiplexed RT-PCR assay for nanopore whole genome sequencing of Tilapia lake virus (TiLV)
Source: Sci Rep. 2023 Nov 20;13:20276. doi: 10.1038/s41598-023-47425-w (PMC10661697; doi:10.1038/s41598-023-47425-w)

3-1-22 (RNA is D1-2) seg1-10.tif

02/03/2022 12:26:0

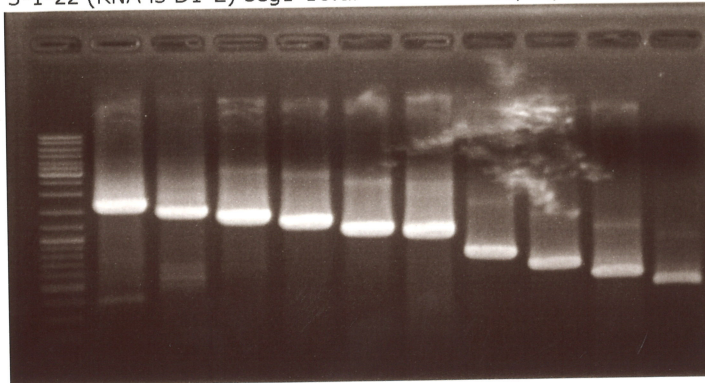

Supplement: Supplementary file 1 — Supplementary Information 1. [file 41598_2023_47425_MOESM1_ESM.pdf]

X

240822 PCR cDNA 10 seg C1.tif

08/24/2022 15:41:4

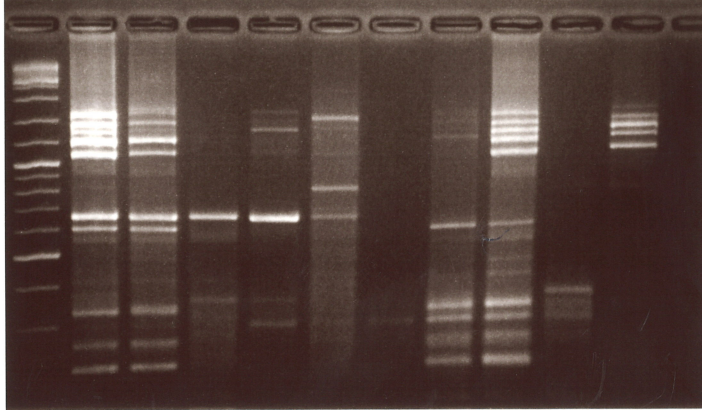

X

150822 PCR cDNA 10 seg C2 10 sample.tif

08/15/2022 12:39:4

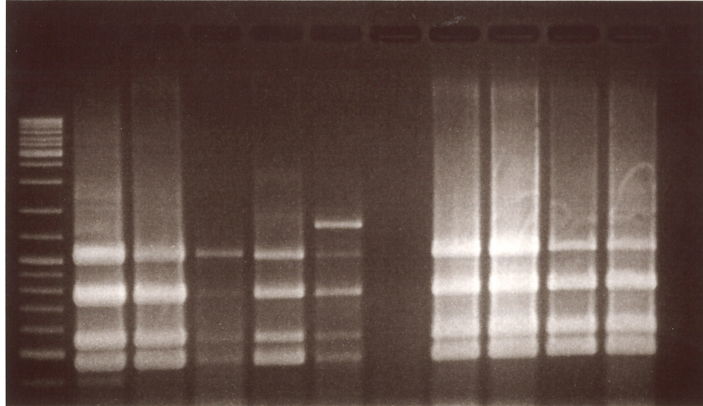

Supplement: Supplementary file 2 — Supplementary Information 2. [file 41598_2023_47425_MOESM2_ESM.pdf]

12-07-21 multiplex of TiLV seg 123458 and 0267202RNA:45n2

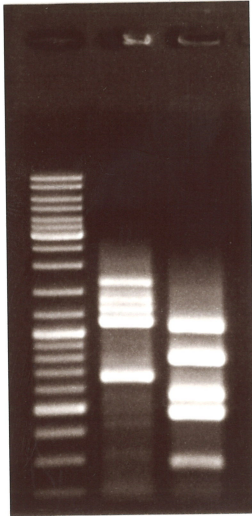

Supplement: Supplementary file 4 — Supplementary Information 4. [file 41598_2023_47425_MOESM4_ESM.pdf]

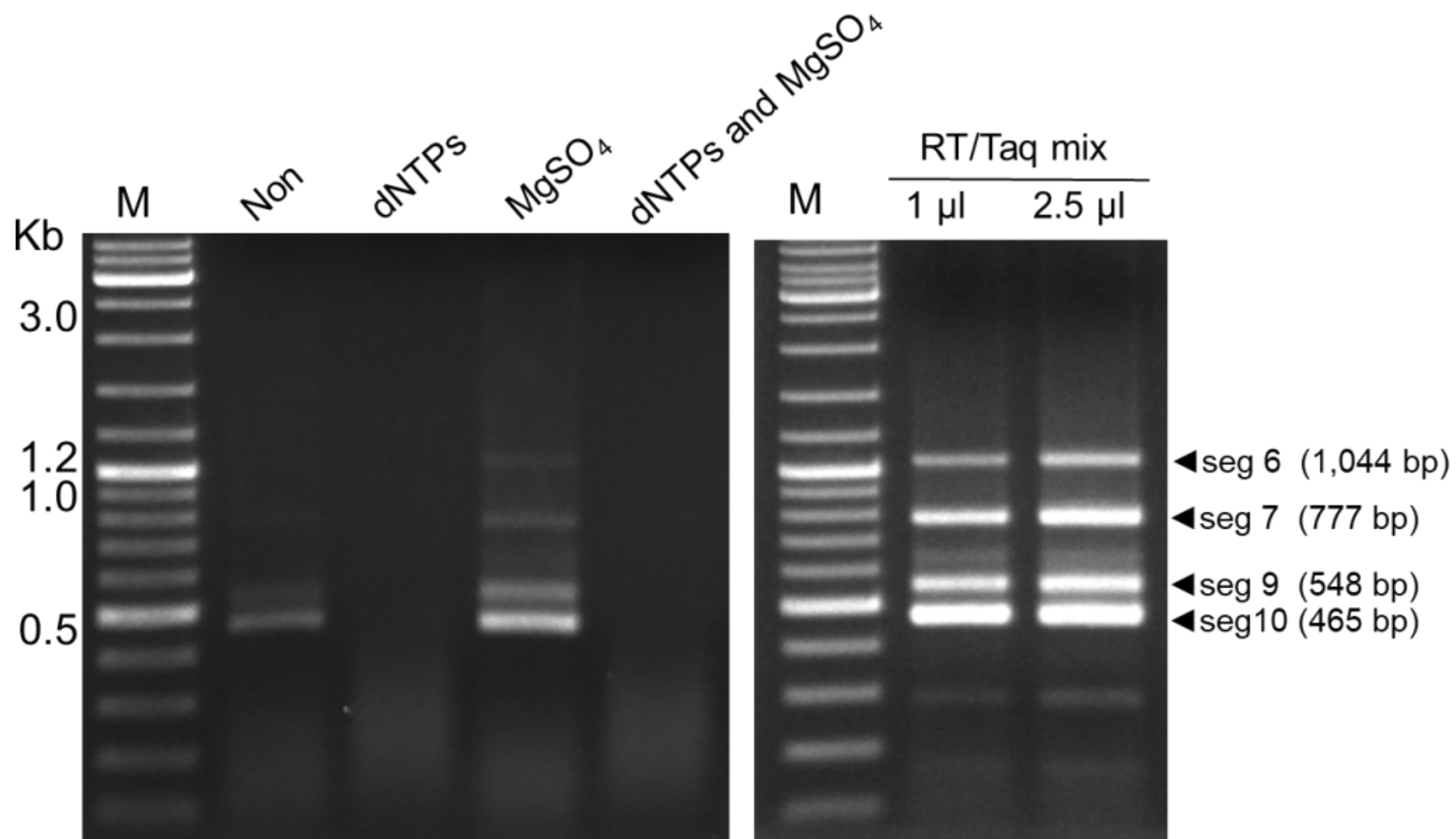

Supplement: Supplementary file 6 — Supplementary Figure 1. [file 41598_2023_47425_MOESM6_ESM.pdf]

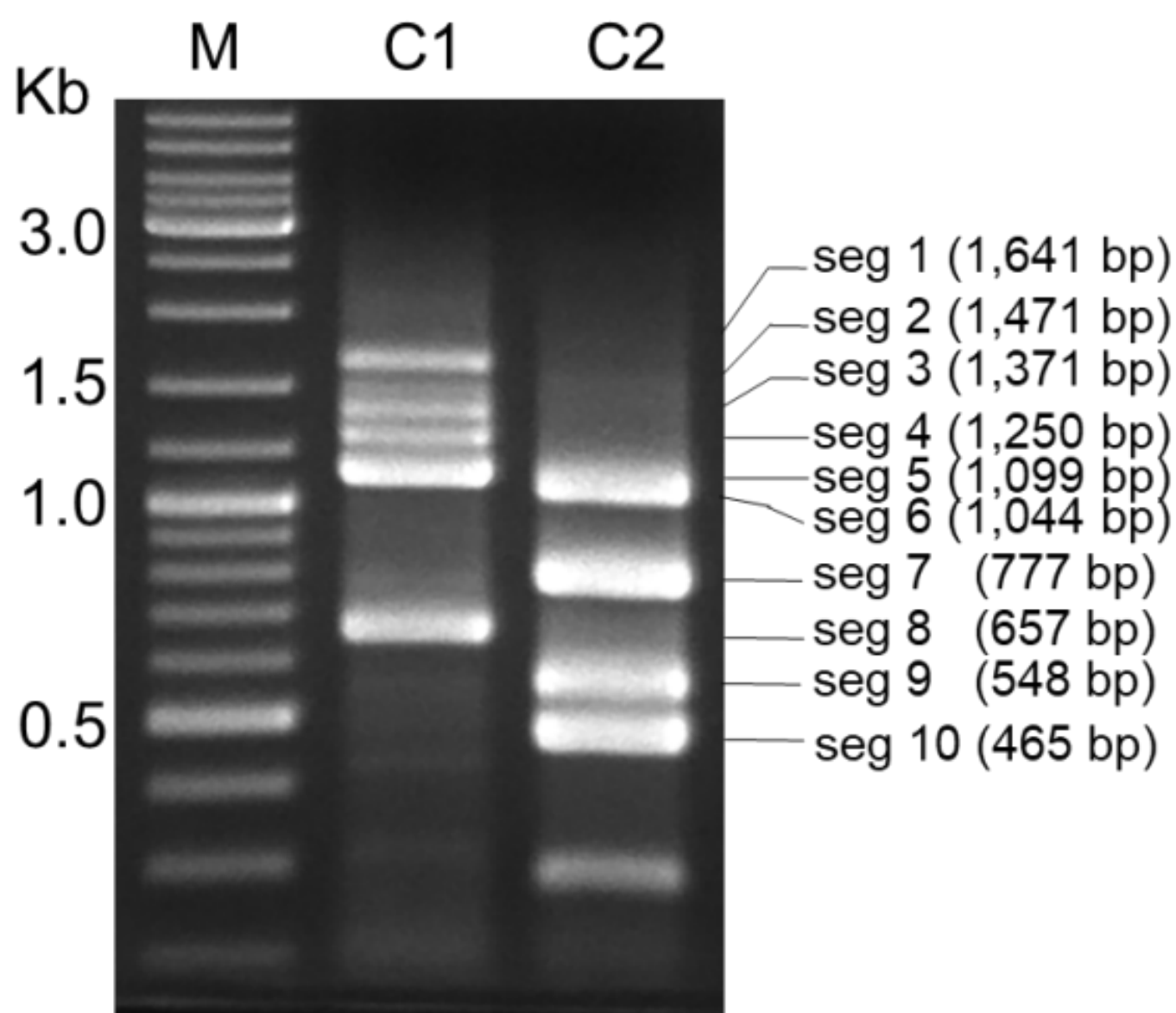

Supplement: Supplementary file 7 — Supplementary Figure 2. [file 41598_2023_47425_MOESM7_ESM.pdf]

# A

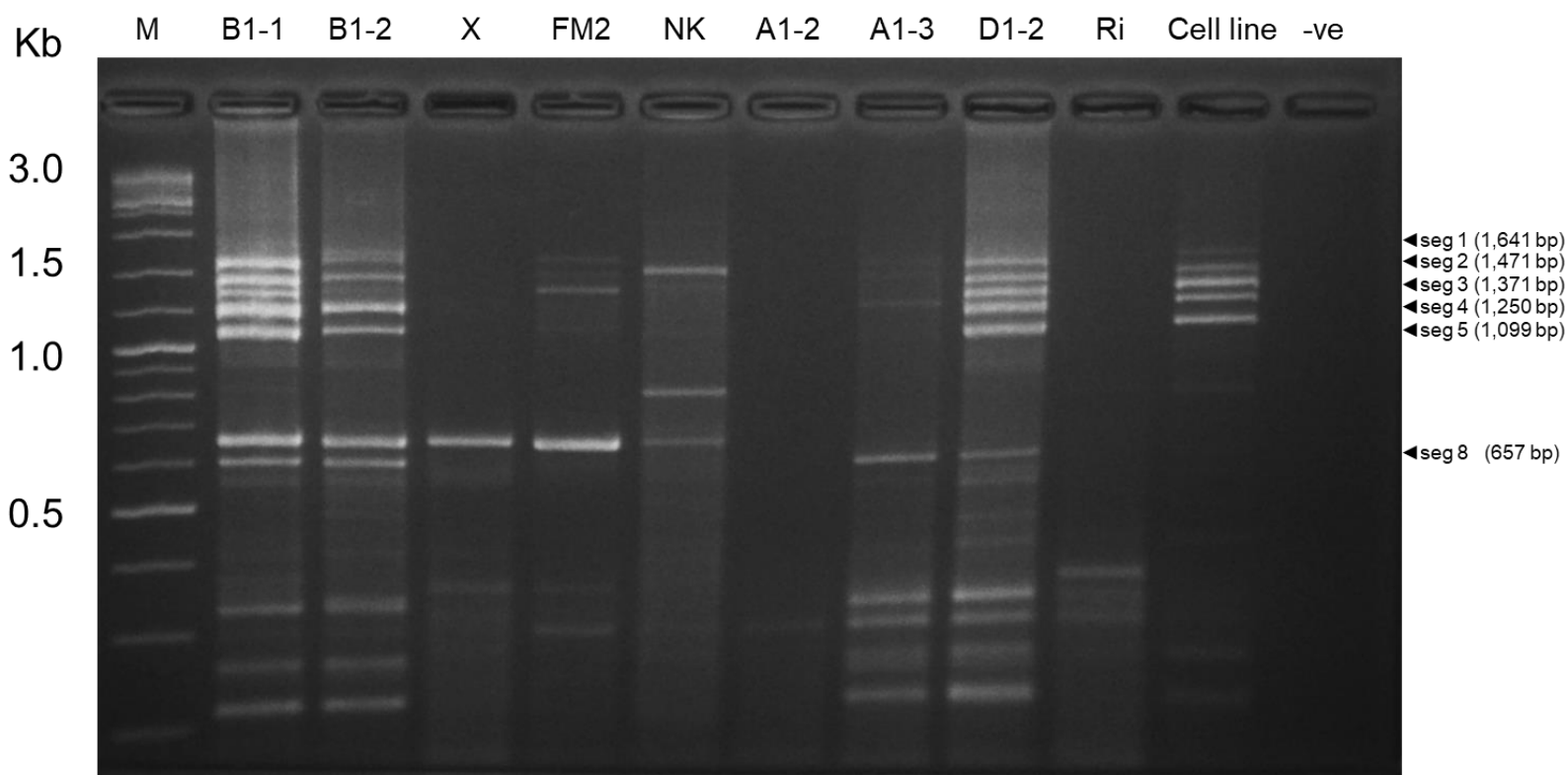

# B

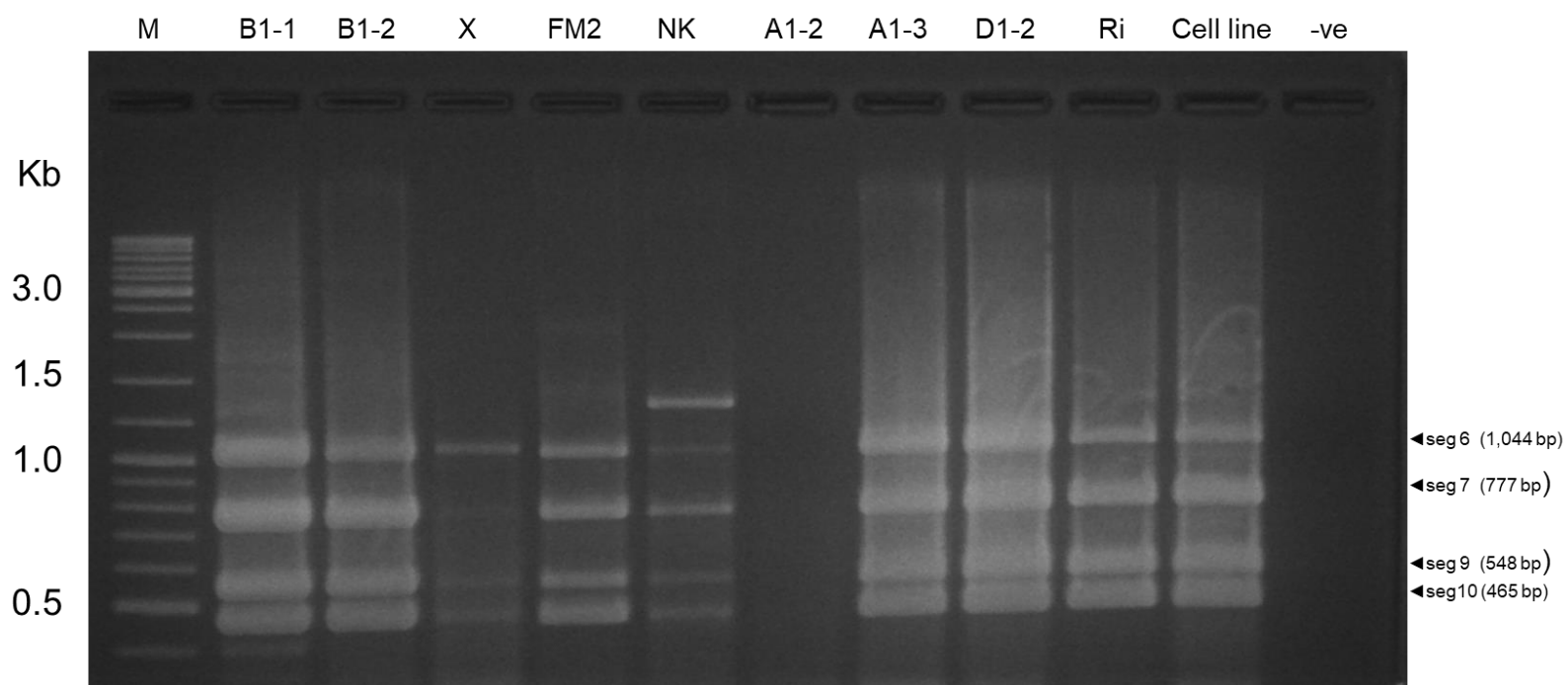

Supplement: Supplementary file 8 — Supplementary Figure 3. [file 41598_2023_47425_MOESM8_ESM.pdf]
