# Supplementary material for: A multiplexed RT-PCR assay for nanopore whole genome sequencing of Tilapia lake virus (TiLV)
Source: Sci Rep. 2023 Nov 20;13:20276. doi: 10.1038/s41598-023-47425-w (PMC10661697; doi:10.1038/s41598-023-47425-w)
Supplement: Supplementary file 5 — Supplementary Information 5. [file 41598_2023_47425_MOESM5_ESM.docx]

**Supplemental Figure 1**. Multiplex RT-PCR condition optimization. The original reaction (Non) and modified reactions with additions of dNTPs, MgSO_4_, dNTPs + MgSO_4_, and RT/Taq enzyme mix were compared. Representative results from mPCR reaction 2 are shown. A DNA marker (New England Biolabs) was used to visualize the PCR products. RNA sample Ri (Table 2) was used in this trial.

**Supplemental Figure 2**. Multiplex RT-PCR amplification of TiLV segments. Two sets of reactions were used to amplify 10 TiLV segments using TiLV RNA templates. Conditions C1 and C2 were used for Reaction 1 and Reaction 2, respectively. C1 amplified segments 1, 2, 3, 4, 5, and 8, while C2 amplified segments 6, 7, 9, and 10. A DNA marker (New England Biolabs) was used to visualize the PCR products. RNA sample Ri (Table 2) was used in this experiment.

**Supplemental Figure 3**. Original gel photo with Lane X which was removed in Figure 2. (see above Figure 2 and its legend).

Legend of Figure 2. Amplification results of multiplex PCR (mPCR) for TiLV segments. Two separate reactions (Reaction#1 and Reaction#2) were used to amplify 10 TiLV segments. Reaction#1 amplified segments 1, 2, 3, 4, 5, and 8 (Fig 2A), while Reaction#2 amplified segments 6, 7, 9, and 10 (Fig 2B). A 2-log DNA marker (New England Biolabs) was used to visualize the PCR products. -ve, no template control. Codes of samples are listed in Table 2. The white dash lines indicate where two images were connected since one lane (Lane X) was excluded from the original gel photo (Supplemental Figure 3).
