# Supplementary material for: A multiplexed RT-PCR assay for nanopore whole genome sequencing of Tilapia lake virus (TiLV)
Source: Sci Rep. 2023 Nov 20;13:20276. doi: 10.1038/s41598-023-47425-w (PMC10661697; doi:10.1038/s41598-023-47425-w)

25-06-21 multiplex of TiLV seg 367910, 3679 [illegible]

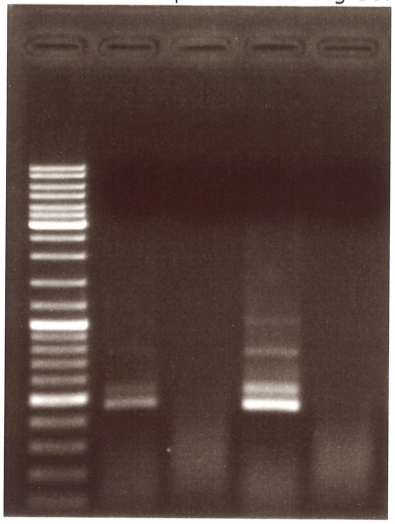

27-06-21 multiplex of TiLV seg 3679 and 10 [illegible]

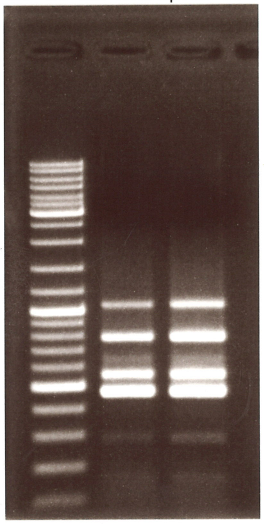

Supplement: Supplementary file 3 — Supplementary Information 3. [file 41598_2023_47425_MOESM3_ESM.pdf]
